# A large Indian family with rearrangement of chromosome 4p16 and 3p26.3 and divergent clinical presentations

**DOI:** 10.1186/s12881-015-0251-5

**Published:** 2015-11-10

**Authors:** Thomas Iype, Vafa Alakbarzade, Mary Iype, Royana Singh, Ajith Sreekantan-Nair, Barry A. Chioza, Tribhuvan M. Mohapatra, Emma L. Baple, Michael A. Patton, Thomas T. Warner, Christos Proukakis, Abhi Kulkarni, Andrew H. Crosby

**Affiliations:** Department of Neurology, Government Medical College, Thiruvananthapuram, Kerala India; Molecular Genetics, RILD Institute, University of Exeter, Royal Devon and Exeter NHS Hospital, Wonford, Exeter, UK; Reta Lila Weston Institute of Neurological Studies, UCL Institute of Neurology, London, UK; Department of Anatomy and Microbiology, Institute of Medical Sciences, Banaras Hindu University, Varanasi, Uttar Pradesh India; Clinical Neuroscience, Royal Free Campus, UCL Institute of Neurology, London, UK; Southwest Thames Regional Genetics Centre, St George’s Healthcare NHS Trust, London, SW17 0RE UK; Human Genetics and Genomic Medicine, Faculty of Medicine, University of Southampton, Southampton, UK; Wessex Clinical Genetics Service, Princess Anne Hospital, Southampton, UK

**Keywords:** Wolf–Hirschhorn syndrome, WHS, Complex rearrangement of chromosome 4p16, 3p deletion syndrome, 4p partial trisomy syndrome, Mental Retardation, Developmental delay

## Abstract

**Background:**

The deletion of the chromosome 4p16.3 Wolf-Hirschhorn syndrome critical region (WHSCR-2) typically results in a characteristic facial appearance, varying intellectual disability, stereotypies and prenatal onset of growth retardation, while gains of the same chromosomal region result in a more variable degree of intellectual deficit and dysmorphism. Similarly the phenotype of individuals with terminal deletions of distal chromosome 3p (3p deletion syndrome) varies from mild to severe intellectual deficit, micro- and trigonocephaly, and a distinct facial appearance.

**Methods and results:**

We investigated a large Indian five-generation pedigree with ten affected family members in which chromosomal microarray and fluorescence in situ hybridization analyses disclosed a complex rearrangement involving chromosomal subregions 4p16.1 and 3p26.3 resulting in a 4p16.1 deletion and 3p26.3 microduplication in three individuals, and a 4p16.1 duplication and 3p26.3 microdeletion in seven individuals. A typical clinical presentation of WHS was observed in all three cases with 4p16.1 deletion and 3p26.3 microduplication. Individuals with a 4p16.1 duplication and 3p26.3 microdeletion demonstrated a range of clinical features including typical 3p microdeletion or 4p partial trisomy syndrome to more severe neurodevelopmental delay with distinct dysmorphic features.

**Conclusion:**

We present the largest pedigree with complex t(4p;3p) chromosomal rearrangements and diverse clinical outcomes including Wolf Hirschorn-, 3p deletion-, and 4p duplication syndrome amongst affected individuals.

**Electronic supplementary material:**

The online version of this article (doi:10.1186/s12881-015-0251-5) contains supplementary material, which is available to authorized users.

## Background

Microscopically visible rearrangements of chromosome 4p16 may result in two separate syndromes: Wolf–Hirschhorn syndrome (WHS) and partial trisomy 4p syndrome. WHS is a well-recognized contiguous gene deletion syndrome associated with prenatal and postnatal growth delay, a typical ‘Greek warrior helmet’ appearance of the nose, microcephaly, and neurological features including seizures, hypotonia and varying degrees of intellectual impairment [1]. In contrast, 4p16 duplication results in partial trisomy 4p syndrome, which gives rise to variable clinical manifestations including intellectual disability, developmental delay and distinctive facial features [2–4].

About 40–45 % of the WHS cases involve an unbalanced translocation and a deletion of 4p as well as partial trisomy of a different chromosome arm [5]. These unbalanced translocations may be *de novo,* or inherited from a parent with a balanced chromosomal rearrangement. Different rearrangements of chromosome 4p causing WHS and partial trisomy 4p syndrome in the same family have been reported [2–4, 6], as have a few reports of isolated unbalanced translocation involving chromosomes 4 and 3 [7–10]. The size of the chromosomal rearrangement and clinical presentations vary in these cases. Terminal and interstitial deletions of the short arm of chromosome 3 comprises another contiguous gene deletion syndrome (3p deletion syndrome), which may also present with a variable clinical phenotype and associated with a number of different causal deletions. The core features of the syndrome typically include cognitive handicap, growth retardation, microcephaly, and facial dimorphism (ptosis, downslanting palpebral fissures and micrognathia), while variable features include congenital heart defects, postaxial polydactyly, cleft palate, renal and intestinal anomalies [11, 12]. In this report, we document a large Indian pedigree in which individuals have inherited varying combinations of an unusual chromosomal translocation involving chromosome 4p16 and a small section of chromosome 3p26.3, resulting in a range of clinical phenotypes including Wolf–Hirschhorn syndrome, 4p partial trisomy- and 3p deletion syndrome.

## Methods and results

### Study approval

The present studies were reviewed and approved by the senior investigators from Banaras Hindu University, Varanasi, UP and Medical College, Trivandrum, Kerala and Royal Devon & Exeter NHS Foundation Trust. All tissue samples were taken with informed consent in accordance with all ethical standards and protocols. The ethical clearance number is EC/847 and EC registration No. ECR/526/INSt/UP2014. All subjects or their guardians provided informed consent prior to their participation in the study and for publishing this research article. Written parental consent also was obtained to publish any images of minors. Written informed consent was obtained to publish the photographs of affected individuals.

#### Clinical report

**Fig. 1:**
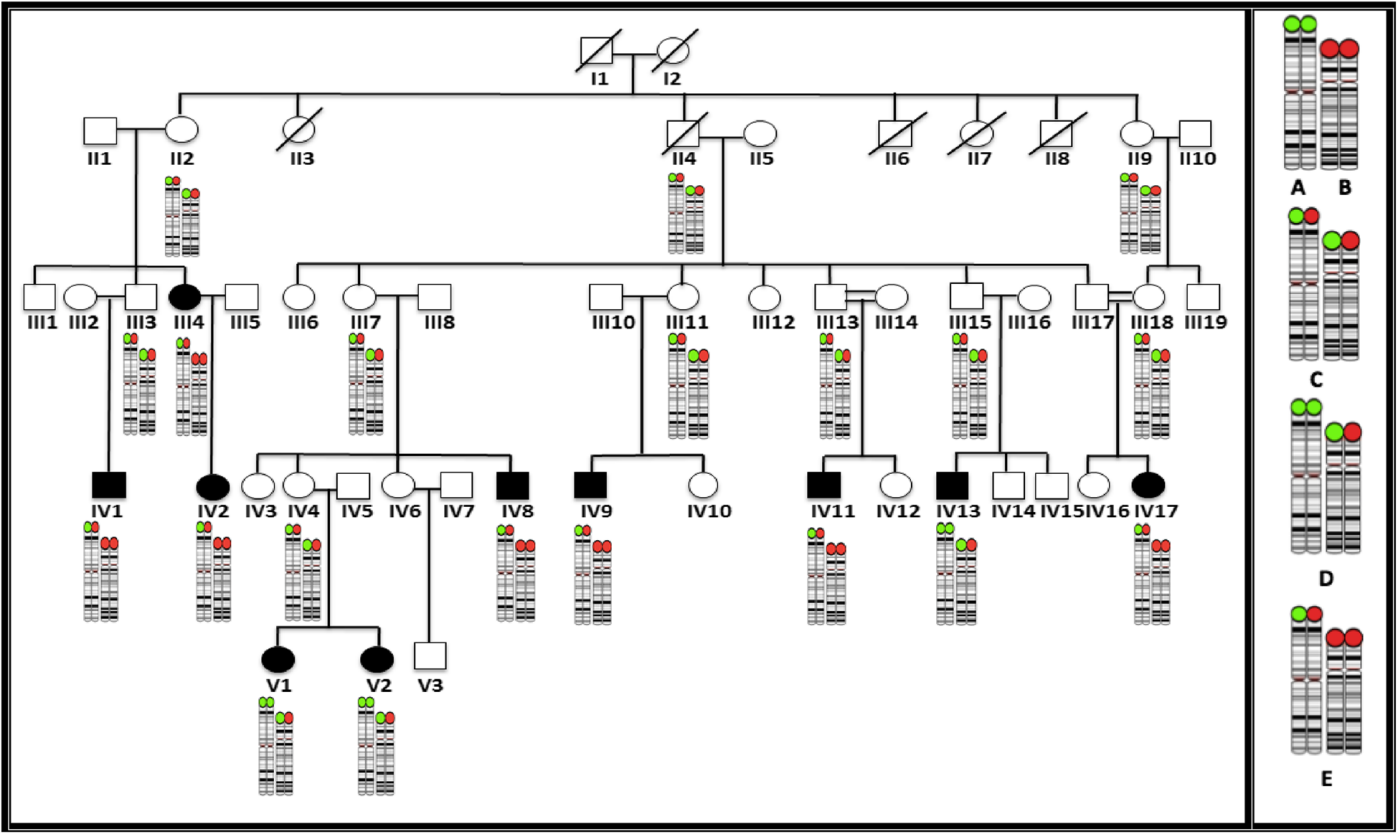
Left panel: family pedigree including an illustration of the chromosome 3 and 4 al rearrangement detected. Right panel: normal chromosome 3 telomere, demarcated with green circle (**a**) and normal chromosome 4 telomere, with red circle (**b**). A balanced translocation outcome is shown in **c**, while unbalanced translocation outcomes are shown in **d** (3p duplication and 4p deletion) and **e** (3p deletion, 4p duplication)

The family, from Kerala (South India), comprises a total of 36 members over five generations (Fig. 1). All 10 affected subjects were evaluated by a neurologist and a clinical geneticist. Pregnancy and delivery of 9/10 affected individuals was uncomplicated, with delivery at gestational age 38 weeks while IV:11 was born pre-term. Patient clinical data and findings are summarized in Tables 1 and 2.

**Table 1:** Clinical presentation of the three patients with the chromosomes 4p16 deletion and 3p26.3 duplication

	V:1	V:2	IV:13
Age	12.5	8.8	12
Gender	F	F	M
Growth Parameters			
Height (cm/SDS^a^)	109 /-5.9	110 /-5.3	128 /-0.3
Head circumference (cm/SDS^a^)	43 /-8.8	42 /-8.0	48 /-4.2
Severe mental retardation	+	+	+
Facial features of WHS::			
• High arched eyebrows	+	+	+
• Broad flat nasal bridge	+	+	+
• Prominent nasal tip	+	+	+
• Prominent eyes	+	+	+
• Ocular hypertelorism	+	+	+
• Short philtrum	+	+	+
• Downturned mouth	+	+	+
• Micrognathia	+	+	+
Seizures	-	+	+
Prenatal growth delay	+	+	+
Hypotonia	+	+	+

^a^ *SDS* standard deviation score (http://www.who.int/childgrowth/standards/en/); above (−2 SDS) normal average

**Table 2:** Clinical presentation of the seven patients with chromosomes 4p16.1 duplication and 3p26.3 microdeletion

Case	III:4	IV:8	IV:9	IV:2	IV:11	IV:1	IV:17
Age	40	20.5	16.7	16.1	14.1	11.7	7.9
Sex	F	M	M	F	M	M	F
Developmental delay	+	+	+	-	+	+	+
Intellectual disability	Severe	Mild	Mild	Mild	Severe	Mild	Mild
Height (cm/SDS)	157/-1.1	179/0.2	172/-0.4	151/-2.0	130/-3.8	143/-0.3	122/-0.4
Head circumference (cm/SDS)	52/-2.5	55/-1.3/Mild	53.5/-1.9/	53/-1.7	49/-4.1/	51/-2.4/	51/-1.5
Shape	NAD	Brachycephaly	Brachycephaly	NAD	Trigonocephaly	Brachycephaly	NAD
Facial features	Prominent supraorbital ridges & glabella; prominent midface; prognathism	Mild prominence supraorbital ridges & glabella	Prominent supraorbital ridges & glabella; prominent midface; mild prognathism	Mild prominence of supraorbital ridges & glabella; prominent midface; prognathism	Hypertelorism, Prominent midface; Flat broad nasal tip, low hanging columella	NAD	NAD
					Highly arched eyebrows		
					Low frontal and nuchal hair line		
Nose	Prominent nasal tip	Prominent nasal tip	Prominent nasal tip	Prominent nasal tip	Broad high nasal bridge	Flat nasal bridge	NAD
					Prominent nasal tip		
Mouth	Thin vermillion of the upper lip; High arched palate; cleft lip; gum hypertrophy	NAD^a^	High arched palate	NAD	Short philtrum	NAD	NAD
					Cleft lip		
Ears	Strabismus	Low set ears	Prominent ears, malformed helix	Prominent, low set ears	Low set ears; malformed helix	Low set ears	NAD
Hand deformity	Campodactyly	Campodactyly	Long digits	NAD	Campodactyly	Clindactily	NAD
Feet deformity	Pes cavus; inversion deformity of feet						
Seizures	+	-^b^	-	-	+	-	-

^a^NAD- no abnormality detected; ^b^- no seizures

V:1 (born in 2000) and V:2 (born in 2004) are the only affected siblings in generation V of the pedigree. Birth weight was 1.5 kg in both cases, length and head circumference at birth were not reported. V:1 was floppy at birth, and developed severe physical and psychomotor developmental delay (sitting at 4 years, currently can only stand with support, delayed or poor eye contact and no speech). A past history of febrile seizures and status epilepticus is reported. Stereotypies of recent onset included rocking and repeated hand washing. Dysmorphic facial features were noted (Table 1; Additional file 1: Figure S1) as well as hand malformations including tapering-long fingers, bilateral inability to abduct the thumb, single transverse palmar crease, hypermobile metacarpophalangeal joints of thumb, overlapping fingers and pes planus. Neurological examination at 12 years of age revealed: increased tone and reflexes in lower limbs. At the age of 12 she developed stereotypic body movements such as bending from side to side, body rocking and hand washing. The younger sibling (V:2) required ventilation for a month post-delivery. Developmental milestones were delayed, walked with support at age 2. Speech is limited to a few words and she is unable to read and write. Generalised tonic-clonic seizures developed at 17 months. Unilateral agenesis of the kidney was seen on ultrasound. Additional clinical features included sacral dimple, overlapping and tapered fingers, flexion contracture of proximal interphalangeal joints, inability to abduct the thumb, simian hand line, hypermobile metocarpophalangeal joint of thumb, rocker bottom feet, prominent talus, medial malleolus, ear deformities (left preauricular sinus, small ears with small lobule, ‘cauliflower’ shaped left ear and pointed pinna). Neurological examination conducted at age 8 revealed left lower motor neuron facial palsy, reduced tone (upper limbs), and increased tone in the lower extremities with exaggerated reflexes. MRI imaging of the brain showed hypo-dense lesions in frontal lobes.

IV:13 is a maternal cousin of siblings V:1 and V:2. His birth weight was 1.8 kg. Developmental concerns were noted early on and progress was similar to V:1. At the age of 2 he had not developed speech and walking, could sit with support and independently roll from back to front. He developed epilepsy with generalized tonic-clonic seizures at 12 months, which is currently under control on phenytoin. He also has swallowing difficulties, drooling and urinary incontinence. He responds to noise, and recognizes his parents. Behavioral problems include self-harm and stereotypies including body rocking movements. Facial features are as described in Table 1 and ear and limb deformities resembling those of V:2. Additional findings included: strabismus, a high arched palate, cleft lip, fixed flexion deformity of the elbows, flat occiput and widely spaced nipples. Neurological examination revealed generalized hypotonia, brisk reflexes and out-turned feet. Ultrasonography revealed kidney malformation, and brain MRI revealed dysgenesis of the corpus callosum.

Individual IV:11 was born pre-term (birth weight 1.2 kg) to consanguineous parents. He had severe developmental delay with no speech and walked with support at age 3. He responds to sounds but hearing function has not been tested. He has a history of surgically treated cleft lip and complex partial seizures. On neurological examination he had gaze evoked nystagmus, limb ataxia, gait ataxia; exaggerated knee and ankle jerks and up-going plantar reflexes. The dysmorphic features are described in Table 2 and Additional file 1: Figure S1. Additional findings on examination were sacral dimple, strabismus and hypospadius. Behavioral abnormalities included stereotypies including rocking, hand wringing, repeated hand washing, head banging and self-harm.

III:4 was the eldest affected child of the third generation. She had no speech or walk until age 2 and has medically resistant secondarily generalized tonic-clonic seizures. At the time of assessment she could not walk unaided, her speech was dysarthric with limited vocabulary and she was unable to read or write. Neurological examination revealed kyphosis, reduced upper limb deep tendon reflexes, absent knee and ankle jerk, hypotonia, scanning speech, gaze evoked nystagmus, and an ataxic gait. Facial features were as described in Table 2. Her daughter, IV:2, started to walk at age 3 and speak at age 4. She displayed aggressive behavior and had mild intellectual impairment. Neurological examination revealed absent knee and ankle jerks and dysarthria. No history of seizures was reported, and distinctive dysmorphic features were noted (Table 2).

IV:17 was born to consanguineous parents. Apart from mild learning difficulties and limited vocabulary, no neurological or other physical abnormalities were detected on examination.

IV:8, IV:9 and IV:1 had delayed walking and speech at 2.5 – 3 years, and similar levels of mild learning difficulties. The birth weight of only IV:8 was documented (2.5 kg). Individual IV:8 could never run due to poor balance, and although he went to a school up to tenth grade, his intellectual abilities are limited. On neurological examination had mild upper and lower motor neuron signs, ataxia and could not stand on his heels. Brain MRI revealed mild cerebral atrophy. Case IV:9 had a previous history of lumbar discectomy. All the observed phenotypic abnormalities are described in Table 2 and Additional file 1: Figure S1.

#### Microarray analysis and flourescent in situ hybridization (FISH)

DNA samples from family members were genotyped using the Illumina HumanCytoSNP-12 v2.1 microarrays to detect copy-number variations (CNVs). The Illumina Infinium HD assay protocol was followed and the array processed on a BeadStation 500G. Image data was processed in the Illumina GenomeStudio software to generate genotype calls, B allele frequency and logR ratio values. These were further analysed for CNVs using Illumina’s KaryoStudio software. This revealed a 3p26.3 (0–2144867)x3duplication and 4p16.3-16.1 (0–10308871)x1 deletion in three affected individuals (IV:13; V:1; V:2), and a reciprocal 3p26.3 (0–2118422)x1deletion and 4p16.3-16.1 (0–10290552)x3duplication in seven affected individuals (III:4; IV:8; IV:9; IV:2; IV:11; IV:1; IV:17), including two (III:4 and IV:11) with more severe phenotype than others (Additional file 1: Table S1, Figure S1 and Figure S2). For FISH, Kreatech probes D4S33060 and D3S4558 were used to define either balanced or unbalanced t(3p;4p) translocations in three individuals, case V:2, IV:9 and III:3 on whom samples were available for study (Additional file 1: Figure S3).

## Discussion and conclusions

We describe a complex reciprocal translocation involving chromosomes 3 and 4 in ten affected family members of a five-generation pedigree, resulting in clinical manifestations of 3 rare chromosomal syndromes. This is, to our knowledge, the first reported case study of a chromosomal imbalance that combines chromosome 4p deletion (WHS), 4p partial trisomy and 3p deletion syndromes in the same family. Nine of the 10 affected family members (II:2; III:7; III:10; III:12; III:14; III:17 and IV:4) were born from a healthy parent with a balanced translocation of chromosomes 3 and 4, while a single affected individual (IV:2) inherited her imbalanced chromosomal complement from her affected mother (III:4) who herself displayed the same 4p16.1 duplication and 3p.26.3 microdeletion.

Deletion of the chromosome 4p region has previously been shown to result in a widely recognized phenotype compared with gains of the same region, which result in more variable clinical manifestations. The family described here includes three family members with chromosome 4p deletion resulting in WHS, all of whom displayed the typical clinical features of this condition. The characteristic phenotype of WHS includes a typical facial appearance, significant intellectual disability and poor growth [13]. The high arched eyebrows and prominent nasal tip that were seen in all three WHS cases described here may relate to the progression of these facial features with age, as has been previously suggested [1]. Similarly, the ear hand and feet anomalies, and dysgenesis of corpus callosum, have previously been documented in WHS cases [1, 14].

At a molecular level, within the terminal 1.9 Mb region on 4p16.3, Wolf-Hirschhorn syndrome candidate region 2 (WHSCR-2) was described as the likely critical region for WHS [15]. Candidate genes in that region have been suggested to cause particular clinical aspects of WHS [16, 17]. Haploinsufficiency of the *WHSC1* gene [OMIM 602952], which is disrupted by the proximal breakpoint of WHSCR-2, has been associated with both facial characteristics and growth delay [18, 19]. The *LETM1* gene, residing entirely within WHSCR-2, is considered to play an important role in the pathogenesis of seizures [20–24]. However, genotype–phenotype correlation analyses suggested that haploinsufficiency of *LETM1* alone may not be sufficient in causing seizures and that loss of the terminal 1.5 Mb region with breakpoint at ~300 kb from the *LETM1* locus, preserving *LETM1*, is critical for the development of a seizure disorder [25]. Large deletions of chromosome 4p16 incorporating both critical regions are typically associated with profound intellectual disability, seizures and psychosis in addition to the typical facial appearance [15, 18, 19]. Our microarray analysis of the three WHS cases described here with 4p16.3-16.1 deletion and 3p26.3 duplication identified a ~5 Mb deletion encompassing WHSCR-2 which resulted in severe developmental delay with absent (2/3) or impaired speech (1/3), absent (2/3) or significantly delayed onset of walking (1/3), as well as generalized tonic-clonic seizures (3/3) and behavioral abnormalities (2/3), consistent with those previously described in WHS.

Compared to the classical presentation seen in the three WHS patients, the seven chromosome 4p partial trisomy patients displayed a much more variable clinical picture. The degree of intellectual deficit present across the seven cases varied from severe (2/7) to mild (4/7). The majority of the seven patients displayed clinical features previously described for trisomy of 4p syndrome, including prominent supraorbital ridges (5/7), prominent nasal tip (5/7), prognathism (3/7), camptodactyly (2/7) and malformed and low-set ears (5/7). Although head circumference was reduced in all seven cases, height was variable ranging from normal (5/7) to short stature (2/7) (Table 2). Overexpression or inactivating mutations of two genes mapping to the chromosomal region involved, fibroblast growth factor receptor 3 (*FGFR3)* and *WHSC1*, have been reported to be associated with overgrowth features such as macrocephaly, campodactyly and tall stature [26, 27]. *FGFR3* is a physiological negative regulator of bone growth, and has been associated with syndromic presentations affecting bone growth including the overgrowth syndrome-CATSHL (campodactyly, tall stature, and hearing loss) [26]. Similarly, overexpression of *WHSC1* has been suggested as a contributor to the overgrowth seen as part of this condition. In mice, haploinsufficency of *WHSC1* resulted in growth retardation [27]. Two of the 7 chromosome 4p duplication cases (III:4 and IV:11) demonstrated discordant clinical manifestations including severe intellectual disability, short stature, microcephaly, generalized tonic-clonic seizures, gum hypertrophy and cerebellar signs, compared with relatively mild learning difficulties in the remaining five cases (IV:8; IV:9; IV:2; IV:1 and IV:17). The cerebellar signs and gum hypertrophy seen in the 2 severely affected cases may relate to long term phenytoin treatment.

The craniofacial features observed in 1 of the 2 partial 4p trisomy cases (IV:11), including high arched eye brows, hypertelorism, trigonocephaly as well as marked reduction in growth milestones and behavioral changes, are not previously recognized in partial 4p trisomy syndrome (Table 2; & Additional file 1: Figure S1). While this may reflect as-yet unrecognized clinical variability in this disorder, it is more likely to be due to the complexity of the chromosome imbalance in these cases.

The chromosome 3pter rearrangement defined here is somewhat smaller compared with rearrangements previously reported as part of chromosome 3p duplication syndrome. The 2.7 Mb chromosome 3 microdeletion detected in this family includes the *CNTN4* and *CHL1* genes, which have previously been shown to have a pleotropic effect and contribute to learning difficulties in affected individuals [28–33]. Further, *CNTN4* deletion has been associated with microcephaly, trigonocephaly, hypertelorism, growth retardation and ear abnormalities. However these clinical features were present in only two of the seven chromosome 3p26.3 microdeletion cases (III:4 & IV:11) presented here. This may mean that other factors including age of examination, other genes in the 3pter region, epigenetic phenomena, expression or regulatory variation and the unmasking of recessive variants residing on the other unperturbed allele, may also be required for the development of these features.

## Availability of supporting data

All the supporting data are included as Additional file 1 and available in the online version of the paper.

## Additional file

Additional file 1:
**Supplemental information includes a table that illustrates DNA copy alterations identified using micro-array copy number analysis, the photos of affected individuals, the illustration of beadarray analysis of SNP data, used to identify the chromosomal subregion duplication and deletion events present in family members, and FISH karyotype analyses.** (DOCX 1037 kb)

## Abbreviations

WHS: Wolf–Hirschhorn syndrome
CNVs: Copy-number variations
WHSCR: Wolf–Hirschhorn syndrome critical regions
CATSHL: Campodactyly, tall stature, and hearing loss
FGFR3: Fibroblast growth factor receptor 3
LETM1: Leucine zipper/EF-hand-containing transmembrane protein 1
