# Supplementary material for: A large Indian family with rearrangement of chromosome 4p16 and 3p26.3 and divergent clinical presentations
Source: BMC Med Genet. 2015 Nov 10;16:104. doi: 10.1186/s12881-015-0251-5 (PMC4641370; doi:10.1186/s12881-015-0251-5)
Supplement: Additional file 1: — Supplemental information includes a table that illustrates DNA copy alterations identified using micro-array copy number analysis, the photos of affected individuals, the illustration of beadarray analysis of SNP data, used to identify the chromosomal subregion duplication and deletion events present in family members, and FISH karyotype analyses. (DOCX 1037 kb) [file 12881_2015_251_MOESM1_ESM.docx]

**SUPPLEMENTAL INFORMATION**

**A large Indian family with rearrangement of chromosome 4p16 and 3p26.3 and divergent clinical presentations**

Thomas Iype ^1*^, Vafa Alakbarzade ^2,3*^, Mary Iype ^1*^, Royana Singh ^4*^, Ajith Sreekantan-Nair ^2^, Barry A Chioza ^2^, Tribhuvan M Mohapatra ^4^, Emma L Baple ^2,7^, Michael A Patton ^2,6^, Thomas T Warner^3^, Christos Proukakis ^5^, Abhi Kulkarni ^6^, Andrew H Crosby ^2^

Affiliations:

^1^Department of Neurology, Government Medical College, Thiruvananthapuram, Kerala, India, beenaiype@gmail.com, tvm_agkoshy@sancharnet.in

^2^Molecular Genetics, RILD Institute, University of Exeter, Royal Devon and Exeter NHS Hospital, Wonford, Exeter, UK, v.alakbarzade@exeter.ac.uk, B.Chioza@exeter.ac.uk

^3^Reta Lila Weston Institute of Neurological Studies, UCL Institute of Neurology, London, UK, vafa.alakbarzade.10@ucl.ac.uk

^4^Department of Anatomy and Microbiology, Institute of Medical Sciences, Banaras Hindu University, Varanasi, Uttar Pradesh, India, tmmohapatra2000@yahoo.com, drroyanasingh@gmail.com

^5^Clinical Neuroscience, Royal Free Campus, UCL Institute of Neurology, London, UK, c.proukakis@ucl.ac.uk

^6^Southwest Thames Regional Genetics Centre, St George's Healthcare NHS Trust, London SW17 0RE, UK, Abhijit.Kulkarni@stgeorges.nhs.uk

^7^Human Genetics and Genomic Medicine, Faculty of Medicine, University of Southampton, Southampton, UK; Wessex Clinical Genetics Service, Princess Anne Hospital, Southampton, UK, E.Baple@exeter.ac.uk

*contributed equally.

Correspondence to: [a.h.crosby@exeter.ac.uk](mailto:a.h.crosby@exeter.ac.uk) or [v.alakbarzade@exeter.ac.uk](mailto:v.alakbarzade@exeter.ac.uk)

Table 1 Supplementary data: DNA copy alterations identified using micro-array copy number analysis.

| **Patient** | **ISCN description** | |
| --- | --- | --- |
| V: 1 |  |  |
| V: 2 | Dup 3p26.3 (0-2161551)x3 | Del 4p16.3-16.1 (0-9919593)x1 |
| IV: 13 |  |  |
| III: 4 |  |  |
| IV: 1 |  |  |
| IV: 2 |  |  |
| IV: 8 | Del 3p26.3 (0-2135106)x1 | Dup 4p16.3-16.1 (0-9901274)x3 |
| IV: 9 |  |  |
| IV: 11 |  |  |
| IV: 17 |  |  |

| 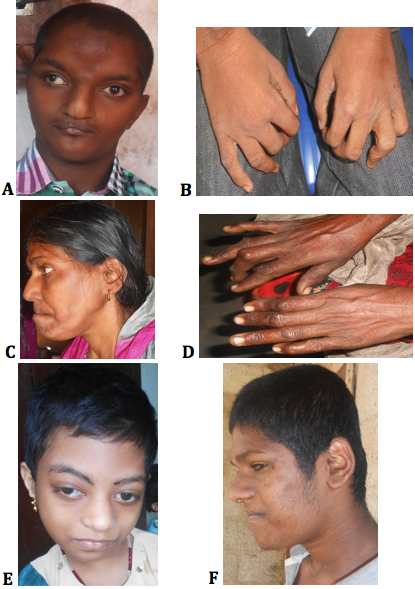 |
| --- |
| Figure 1 Supplementary data. (A)(B) case IV: 11; (C)(D) case III: 4; (E) case V: 1; (F) case IV: 9. Written consent from all individuals depicted in the photographs was obtained to publish the images. |

|  | Figure 2.Supplementary data. Example of beadarray analysis of SNP data, used to identify the chromosomal subregion duplication and deletion events present in family members. A: Chromosome 3p26.3 gene content (i) deletion (ii) and duplication (iii). B: Chromosome 4p16.3-16.1 gene content (i), deletion (ii) and duplication (iii). |  | |
| --- | --- | --- | --- |
| 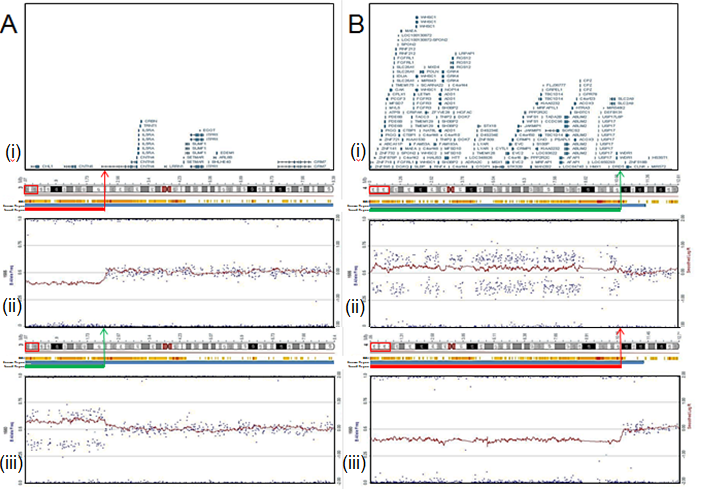 | | |  |

| 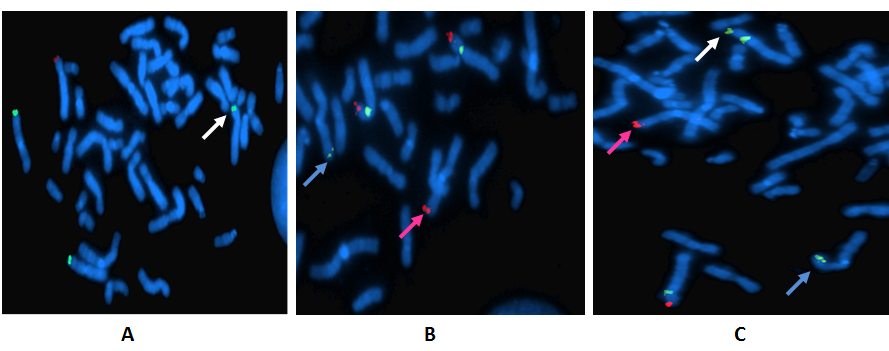 |
| --- |
| Figure 3.Supplementary data. FISH karyotype analyses. Kreatech probe D4S33060 (red) was used for 4p16.3 or 4pter and Kreatech probe D3S4558 (green) was used for 3pter. FISH karyotype analyses for; (A) case V:2 with 4p16.3-16.1 deletion and 3p26.3 duplication with probes for chromosome 3p (red) and 4p (green). Derivative chromosome 4 demarcated with white arrow, (B) case IV:9 with 4p16.3-16.1 duplication and 3p26.3 deletion, with 4p centromere marker (green), 4pter marker (red) and 3pter marker (also green, so indicated by blue arrow). The derivative chromosome 3 is demarcated with a pink arrow. (C) Case III:3 with balanced translocation between 4p16.3-16.1 and 3p26.3, with probes for 4p centromere (green), 4pter (red) and 3pter (also green, so indicated by blue arrow). The derivative chromosome 4 is indicated with a white arrow, and derivative chromosome 3 indicated with a pink arrow. |
